# Associations Between Amantadine Usage, Gait, and Cognition in PSP: A *post-hoc* Analysis of the Davunetide Trial

**DOI:** 10.3389/fneur.2020.606925

**Published:** 2020-12-21

**Authors:** Marian L. Dale, Barbara H. Brumbach, Adam L. Boxer, Amie L. Hiller

**Affiliations:** ^1^Department of Neurology, Oregon Health & Science University, The VA Portland Health Care System, Portland, OR, United States; ^2^Biostatistics and Design Program, Oregon Health & Science University, Portland, OR, United States; ^3^Department of Neurology, University of California, San Francisco, San Francisco, CA, United States

**Keywords:** progressive supranuclear palsy, amantadine, gait, balance, cognition

## Abstract

**Introduction:** Amantadine anecdotally improves gait in progressive supranuclear palsy (PSP) but definitive data is lacking. We investigated associations between amantadine usage, gait, cognition, and activities of daily living in 310 subjects with PSP using data from the davunetide trial.

**Method:** We compared baseline demographics, PSP Rating Scale (PSPRS), Repeat Battery for the Assessment of Neuropsychological Status (RBANS), and Schwab and England Activities of Daily Living (SEADL) scores between subjects taking vs. not taking amantadine using chi-square tests for categorical variables and independent sample *t*-tests for continuous variables. Using the general linear model (GLM), we tested whether group status predicted total PSPRS, PSPRS-gait and midline, total RBANS, RBANS-attention, and SEADL before and after the 52-weeks follow-up.

**Results:** Subjects taking vs. not taking amantadine were similar at baseline, except subjects taking amantadine had a higher Clinical Global Impression (CGI) Score (*p* = 0.01). However, the CGI change score did not differ between groups at week 52 (*p* = 0.10). Using GLM models (controlling for covariates), we found that subjects taking vs. not taking amantadine did not significantly predict total PSPRS, PSPRS-gait and midline, total RBANS, RBANS-attention, or SEADL at baseline, week 52, or the change score between baseline and week 52.

**Discussion:** This *post-hoc* analysis of the davunetide trial did not find an association between amantadine and gait or cognitive measures in PSP, but was not powered to find such a difference. Future studies should still examine amantadine for symptomatic benefit in multiple PSP subtypes.

## Introduction

There are limited pharmacological options available for gait impairment and falls in progressive supranuclear palsy (PSP), but movement disorder clinicians anecdotally note that patients with PSP report fewer falls when taking amantadine. Some clinicians report improved freezing of gait in patients with PSP on amantadine; others suspect improved attention may reduce falls. Other than anecdotal use of amantadine, there are no established pharmacological interventions for postural instability or falls PSP. Levodopa can be helpful for gait to the degree that it improves concurrent bradykinesia (particularly in the PSP-parkinsonism subtype) (1), but it does not improve postural instability (2, 3).

Several retrospective studies have examined amantadine as a treatment for PSP, but none specifically assessed changes in gait or balance. In a retrospective case review of 14 patients with PSP, Rajput found 43% showed improved bradykinesia or rigidity on amantadine, but gait and balance were not specifically assessed (4). In a retrospective review of 16 patients with PSP, Jackson et al. found that of seven patients treated with amantadine, only one displayed improved parkinsonian symptoms overall, and six either showed no change or worsened (5). Kompoliti et al. examined clinical records of 12 autopsy-confirmed PSP cases and found that five of 12 patients received amantadine (6). Two of those five demonstrated modest improvement of parkinsonism (both patients) and neck dystonia (one patient). Three of the five amantadine-treated patients complained of overall deterioration (reported side effects included orthostasis and hallucinations or delusions) (6). In a larger and more promising retrospective review, Niefort and Golbe calculated risk/benefit ratios for various pharmacologic treatments in 87 patients PSP and found that carbidopa-levodopa and amantadine gave the best risk/benefit ratios as monotherapy (0.61 and 0.80, respectively) (7). Other agents studied included MAO-B inhibitors, TCAs, and SSRIs. Overall, existing retrospective reviews of amantadine in PSP are mixed and find that a subset of patients' parkinsonian symptoms respond to amantadine.

Two small studies have prospectively examined the role of amantadine for freezing of gait in PSP. Using a crossover design, Kondo found a significant reduction in freezing of gait on the Freezing of Gait Questionnaire in two subjects with PSP when taking amantadine (8). In another prospective study of 200 mg amantadine IV given twice daily for 2 days, 1 PSP subject showed a mild improvement in freezing of gait, but the other did not respond (9). The effect of amantadine on gait and balance in PSP has not been systematically examined in a large, prospective trial.

As an initial step toward more definitively answering this clinically relevant question, the davunetide trial offers a large, longitudinal dataset in which the relationship between amantadine usage, gait, and cognition in PSP can be examined. The davunetide trial was a randomized, double-blind, placebo-controlled phase 2/3 study of a microtubule stabilizer and reducer of tau phosphorylation (10). It did not achieve its primary endpoints on the PSP Rating Scale (PSPRS) or the Schwab and England Activities of Daily Living (SEADL), but provided the opportunity to examine the natural history of PSP, including motoric, cognitive, imaging, and CSF biomarkers. Information regarding subjects' amantadine status (*taking* or *not taking* and average dose at baseline) is also available within the dataset. We hypothesized that PSP subjects taking amantadine during the davunetide trial may have increased attention and improved gait and balance compared to subjects not on amantadine, and sought to analyze this in a *post-hoc* review of the davunetide dataset.

## Methods

We obtained deidentified longitudinal participant data from baseline through week 52 in 313 randomized PSP subjects in the davunetide trial. We excluded three subjects who were not on amantadine for the duration of the trial or subjects who didn't have data for both baseline and week 52 timepoints, leaving 310 subjects for this analysis. We calculated the average daily total dose of amantadine for the group at baseline. Subjects met PSP criteria from the national Neuroprotection and Natural History in Parkinson Plus Syndromes (“NNIPPS”) Study (11), as the 2017 MDS PSP criteria were not available at the time of recruitment. This analysis was approved by the Oregon Health and Science University Investigational Review Board and was not classified as human research due to the deidentified nature of the data (IRB # 20472).

For *taking* and *not taking* amantadine groups, baseline descriptive data analyzed included age, sex, Schwab and England Activities of Daily Living Scale (SEADL) (12), Clinical Global Impression (CGI) (13), Geriatric Depression Scale (GDS) (14), PSP Rating Scale (PSPRS) (15), and the Repeatable Battery for Neuropsychological Status (RBANS) (16). All variables used in analyses were approximately normal for both groups. To determine if there were group differences at baseline, chi-square tests were used for categorical variables and independent sample *t*-tests for continuous variables.

We then analyzed group differences in SEADL, CGI, Clinical Global Impression-Change (CGI-C), GDS, PSPRS, and RBANS outcomes as well as component scores from the RBANS and PSPRS over the 52-weeks study, including change scores. The RBANS is a cognitive battery that includes with five domains: immediate memory, attention, visuospatial/constructional, language, and delayed memory (16). Total scores range from 40 to 160, and lower scores indicate more severe cognitive deficit. The PSPRS is a global scale of PSP severity and includes subsections for history, mentation, bulbar exam, oculomotor exam, limb motor exam, and gait and midline exam (15). Higher scores on the PSPRS correspond to more severe PSP symptoms (maximum score 100). Within the RBANS we extracted the attention sub-score for further analysis, and within the PSPRS we extracted the gait and midline sub-score. The attention score comprises digit span and coding and the gait and midline sub-score tests the following five measures: neck rigidity and dystonia, arising from a chair, gait, postural stability, and sitting down. Finally, we used the general linear model (GLM) to assess whether there were differences between amantadine groups in RBANS total, RBANS-attention, PSPRS total, PSPRS-gait and midline, and SEADL scores while accounting for possible covariates. Covariates included age, disease duration, davunetide vs. placebo group status, baseline CGI scores, and baseline scores of the respective dependent variable for the 52 weeks and change scores.

## Results

Of 310 subjects analyzed, 43 patients were on amantadine; 267 were not. Among the 43 patients who were on amantadine at the start of the study the average daily dose was 227.9 mg (SD 95.3). Eight of the 43 subjects on amantadine were on a baseline dose of 100 mg or less. Only 10 of the subjects on amantadine were noted to have PSP with parkinsonian features; the majority were considered to have classic Richardson syndrome.

At baseline, taking vs. not taking amantadine groups did not significantly differ on demographics such as age, sex, activities of daily living (SEADL), and depression scores (GDS). The only notable group difference was that the group on amantadine had a higher CGI score at baseline, indicative of more severe illness (Table 1). Taking and not taking amantadine group differences within the davunetide and placebo groups were also examined at week 52 for SEADL, CGI, CGI-C, PSPRS total, RBANS total, PSPRS total change score, and RBANS total change score (Table 1). There were no statistically significant group differences.

**Table 1:** Baseline demographics of subjects in the davunetide trial in taking and not taking amantadine groups.

	**Taking amantadine**									**Not taking amantadine**									**Amantadine vs. no amantadine for total**
	**Total**			**Control**			**Davunetide treatment**			**Total**			**Control**			**Davunetide treatment**			***P***
	***N*** **(%)**			***N*** **(%)**			***N*** **(%)**			***N*** **(%)**			***N*** **(%)**			***N*** **(%)**			
**Sex**																			0.16
Female	16 (37%)			6 (26%)			10 (50%)			130 (49%)			65 (49.6%)			65 (48%)			
Male	27 (63%)			17 (74%)			10 (50%)			137 (51%)			66 (50.3%)			71 (52%)			
**Disease Duration**																			0.29
<5 yrs	40 (95%)			21 (91%)			19 (100%)			229 (90%)			108 (91%)			121 (90%)			
5 yrs+	2 (5%)			2 (9%)			0 (0%)			25 (10%)			11 (9%)			14 (10%)			
	**N**	**M (SD)**	**95%CI**	**N**	**M (SD)**	**95%CI**	**N**	**M (SD)**	**95%CI**	**N**	**M (SD)**	**95%CI**	**N**	**M (SD)**	**95%CI**	**N**	**M (SD)**	**95%CI**	
Age	43	66.7 (6.1)	64.9, 68.5	23	67.4 (7.3)	64.4, 70.4	20	65.9 (4.5)	63.9, 67.9	267	67.8 (6.7)	67.0, 68.6	131	67.2 (6.9)	66.0, 68.4	136	68.4 (6.4)	67.3, 69.5	0.31
SEADL (baseline)	42	4.9 (1.9)	4.3, 5.5	23	4.8 (1.9)	4.0, 5.6	19	5.1 (2.0)	4.1, 6.0	264	5.2 (2.2)	4.9, 5.5	131	5.4 (2.2)	5.0, 5.8	133	5.0 (2.2)	4.6, 5.4	0.42
SEADL (week 52)	34	3.6 (1.8)	3.0, 4.2	18	3.6 (1.9)	2.7, 4.5	16	3.6 (1.8)	2.7, 4.5	202	3.7 (2.2)	3.4, 4.0	100	3.9 (2.4)	3.4, 4.4	102	3.6 (2.0)	3.2, 4.0	0.77
CGI (baseline)	42	4.3 (0.6)	4.1, 4.5	23	4.3 (0.7)	4.0, 4.6	19	4.2 (0.6)	3.9, 4.5	264	3.9 (0.9)	3.8, 4.0	131	3.8 (0.9)	3.7, 4.0	133	3.9 (1.0)	3.8, 4.1	***0.01***
CGI (week 52)	34	5.0 (0.9)	4.7, 5.3	18	4.8 (0.9)	4.4, 5.3	16	5.3 (0.9)	4.8, 5.7	202	4.7 (1.0)	4.6, 4.9	100	4.7 (1.0)	4.5, 4.9	102	4.7 (1.0)	4.5, 4.9	0.10
CGIC (week 52)	33	5.5 (1.0)	5.2, 5.8	18	5.3 (0.9)	4.9, 5.8	15	5.7 (1.0)	5.1, 6.2	201	5.4 (0.9)	5.3, 5.5	99	5.4 (1.0)	5.2, 5.6	102	5.4 (0.9)	5.3, 5.6	0.63
GDS (baseline)	42	13.6 (6.7)	11.6, 15.7	23	14.7 (6.7)	11.9, 17.5	19	12.4 (6.6)	9.4, 15.4	263	12.7 (6.7)	11.8, 13.5	131	12.9 (6.8)	11.7, 14.1	132	12.4 (6.7)	11.3, 13.5	0.38
PSPRS total (baseline)	42	41.6 (9.9)	38.6, 44.6	23	40.8 (8.0)	37.6, 44.1	19	42.6 (12.1)	37.1, 48.0	263	39.5 (11.4)	38.2, 40.9	131	39.2 (11.2)	37.3, 41.1	132	39.9 (11.6)	37.9, 41.9	0.26
PSPRS total (week 52)	34	51.8 (12.6)	47.5, 56.0	18	49.8 (11.2)	44.6, 55.0	16	54.0 (14.1)	47.1, 60.9	204	49.9 (14.2)	48.0, 51.9	103	49.7 (14.4)	46.9, 52.5	101	50.2 (14.1)	47.5, 53.0	0.48
RBANS total (baseline)	42	139.5 (36.8)	128.4, 150.7	23	141.9 (38.9)	126.0, 157.9	19	136.7 (34.9)	120.9, 152.5	259	140.8 (34.1)	136.6, 145.0	129	141.0 (32.5)	135.4, 146.7	130	140.6 (35.7)	134.4, 146.8	0.83
RBANS total (week 52)	31	126.3 (39.8)	112.2, 140.4	17	123.6 (43.9)	102.6, 144.6	14	129.6 (35.6)	110.8, 148.3	183	126.0 (42.2)	119.8, 132.1	92	122.0 (43.3)	113.1, 130.9	91	130.0 (41.1)	121.5, 138.5	0.97

*SEADL, CGI, GDS, PSPRS, and RBANS scores for all groups at baseline and at week 52*.

*Chi-square tests were used for categorical variables and independent sample t-tests for continuous variables*.

Using the GLM we then assessed the following outcome variables: RBANS total, RBANS-attention, PSPRS total, PSPRS-gait and midline, and SEADL scores while controlling for covariates (age, disease duration, davunetide vs. placebo group status, baseline CGI scores, and baseline scores of the respective dependent variable for the 52 weeks and change scores). The primary predictor variable was whether or not someone was taking amantadine. Models were evaluated for each outcome variable at baseline, week 52, and the change score between baseline and week 52. Whether or not someone was taking amantadine did *not* significantly predict any of the outcome variables (RBANS total, RBANS-attention, PSPRS total, PSPRS-gait and midline scores, or SEADL), at baseline, week 52, or in the change score.

## Discussion

This *post-hoc* analysis of the davunetide trial failed to show significant associations between amantadine use and indices of gait and cognitive impairment in PSP. Nonetheless, this is the first analysis of the role of amantadine in a large, longitudinal dataset in PSP. Prior studies examining amantadine and freezing of gait in PSP showed mixed results and were limited to small sample sizes (8, 9). No large studies focusing on the potential role of amantadine for gait and cognition in PSP have been conducted.

The davunetide trial was not powered to find a group difference by amantadine status, and the dataset has several other important limitations. We lacked statistical power to analyze outcomes by baseline or cumulative amantadine dosage. The subjects taking amantadine may have had more severe disease at baseline as reflected in their CGI ratings. We did not have information on fall frequency or the geographic distribution of subjects taking vs. not taking amantadine. Also, the available cognitive and gait data lacked certain important elements. There was no measure of impulsivity in the cognitive data, a feature that clinicians know increases fall risk in PSP. There is also no specific measure of freezing of gait in the PSPRS, and this is relevant because some clinicians note that freezing of gait responds to amantadine in a subset of patients with PSP. Because this trial was conducted prior to establishment of the 2017 Movement Disorders Society PSP Criteria, the dataset lacks sufficient information for statistical analysis of the potential utility of amantadine in various PSP-subtypes.

Based on its purported mechanism of action, there may still be reason to power a larger study to explore the role of amantadine in gait and cognition in PSP. In Parkinson's disease, amantadine may modulate dopamine neurotransmission by promoting dopamine release and inhibiting uptake (17). This dopaminergic effect may benefit the subset of PSP patients with bradykinesia, such as in PSP-parkinsonism. Amantadine's NMDA-receptor antagonist properties account for its role in awareness and attention in diseases such as traumatic brain injury (18–20), ADHD, (21–24), catatonia, (25–28) multiple sclerosis (29), and reduced consciousness (30–34). Reduction of bradykinesia and improvement in awareness would both conceptually improve gait and balance. Finally, there is evidence that amantadine inhibits microglial activation and decreases neural inflammation (35), potentially improving the overall disease course in neurodegenerative disorders such as PSP.

This first analysis of amantadine in a large, longitudinal dataset in PSP failed to show significant associations between amantadine use and indices of gait and cognitive impairment in PSP, but was constrained by the methodological limitations of a *post-hoc* analysis. This analysis did not specifically compare subjects before and after the initiation of amantadine. It is possible that amantadine provides a symptomatic benefit after initiation that later wanes with chronic treatment. Additionally, the majority of subjects in the davunetide trial met 2009 criteria for PSP-Richardson syndrome. Amantadine may still be a beneficial symptomatic treatment for the other subtypes of PSP delineated in the 2017 MDS criteria. Future studies should compare symptomatic benefit before and after amantadine initiation in multiple PSP subtypes.

## Data Availability Statement

The raw data supporting the conclusions of this article will be made available by the authors, without undue reservation.

## Ethics Statement

The studies involving human participants were reviewed and approved by Oregon Health & Science University Investigational Review Board. Written informed consent for participation was not required for this study in accordance with the national legislation and the institutional requirements.

## The AL-108-231 Study Group

*Australia:* David Williams; *Canada:* Anne Louise Lafontaine, Connie Marras, Mandar Jog, Michael Panisset, Anthony Lang, Lesley Parker, Alistair J. Stewart; *France:* Jean-Christophe Corvol, Jean-Philippe Azulay, Philippe Couratier; *Germany:* Brit Mollenhauer, Stefan Lorenzl, Albert Ludolph, Reiner Benecke, Gunter Hoglinger, Axel Lipp, Heinz Reichmann, Dirk Woitalla; *United Kingdom*: Dennis Chan, Adam Zermansky, David Burn, Andrew Lees; *Israel:* Illana Gozes, *United States:* Adam Boxer, Bruce L. Miller, Iryna V. Lobach, Erik Roberson, Lawrence Honig, Edward Zamrini, Rajesh Pahwa, Yvette Bordelon, Erika Driver-Dunkley, Stephanie Lessig, Mark Lew, Kyle Womack, Brad Boeve, Joseph Ferrara, Argyle Hillis, Daniel Kaufer, Rajeev Kumar, Tao Xie, Steven Gunzler, Theresa Zesiewicz, Praveen Dayalu, Lawrence Golbe, Murray Grossman, Joseph Jankovic, Scott McGinnis, Anthony Santiago, Paul Tuite, Stuart Isaacson, Julie Leegwater-Kim, Irene Litvan, David S. Knopman, Lon S. Schneider, Rachelle S. Doody, Lawrence I. Golbe, Erik D. Roberson, Mary Koestler, Clifford R. Jack, Jr., Viviana Van Deerlin, Christopher Randolph, Steve Whitaker, Joe Hirman, Michael Gold, Bruce H. Morimoto.

## Author Contributions

MD: organization and execution of the research project and writing of the first draft of the manuscript. BB: design, execution, review and critique of the statistical analysis, and review and critique of the manuscript preparation. AB and AH: conception of the research project and review and critique of the manuscript preparation. All authors contributed to the article and approved the submitted version.

## Conflict of Interest

AH received funding from Adamas Pharmaceuticals for an investigator-initiated study looking at the effects of extended release amantadine (Gocovri) on falls and gait measures in persons with Parkinson's disease. The remaining authors declare that the research was conducted in the absence of any commercial or financial relationships that could be construed as a potential conflict of interest.

## Abbreviations

CGI: Clinical Global Impression
CGI-C: Clinical Global Impression-Change
GDS: Geriatric Depression Scale
PSPRS: PSP Rating Scale
PSPRS-gait and midline: PSP Rating Scale gait and midline subscore
RBANS: Repeat Battery for the Assessment of Neuropsychological Status
RBANS-attention: RBANS attention subscore
SEADL: Schwab and England Activities of Daily Living.
